# The Insulin Response to Oral Glucose in GIP and GLP-1 Receptor Knockout Mice: Review of the Literature and Stepwise Glucose Dose Response Studies in Female Mice

**DOI:** 10.3389/fendo.2021.665537

**Published:** 2021-05-27

**Authors:** Bo Ahrén, Yuichiro Yamada, Yutaka Seino

**Affiliations:** ^1^ Department of Clinical Sciences Lund, Lund University, Lund, Sweden; ^2^ Department of Endocrinology, Diabetes and Geriatric Medicine, Graduate School of Medicine, Akita University, Akita, Japan; ^3^ Kansai Electric Power Hospital, Osaka, Japan

**Keywords:** GLP-1, GIP, knockout mice, glucose tolerance, insulin

## Abstract

A key factor for the insulin response to oral glucose is the pro-glucagon derived incretin hormone glucagon-like peptide-1 (GLP-1), together with the companion incretin hormone, glucose-dependent insulinotropic polypeptide (GIP). Studies in GIP and GLP-1 receptor knockout (KO) mice have been undertaken in several studies to examine this role of the incretin hormones. In the present study, we reviewed the literature on glucose and insulin responses to oral glucose in these mice. We found six publications with such studies reporting results of thirteen separate study arms. The results were not straightforward, since glucose intolerance in GIP or GLP-1 receptor KO mice were reported only in eight of the arms, whereas normal glucose tolerance was reported in five arms. A general potential weakness of the published study is that each of them have examined effects of only one single dose of glucose. In a previous study in mice with genetic deletion of both GLP-1 and GIP receptors we showed that these mice have impaired insulin response to oral glucose after large but not small glucose loads, suggesting that the relevance of the incretin hormones may be dependent on the glucose load. To further test this hypothesis, we have now performed a stepwise glucose administration through a gastric tube (from zero to 125mg) in model experiments in anesthetized female wildtype, GLP-1 receptor KO and GIP receptor KO mice. We show that GIP receptor KO mice exhibit glucose intolerance in the presence of impaired insulin response after 100 and 125 mg glucose, but not after lower doses of glucose. In contrast, GLP-1 receptor KO mice have normal glucose tolerance after all glucose loads, in the presence of a compensatory increase in the insulin response. Therefore, based on these results and the literature survey, we suggest that GIP and GLP-1 receptor KO mice retain normal glucose tolerance after oral glucose, except after large glucose loads in GIP receptor KO mice, and we also show an adaptive mechanism in GLP-1 receptor KO mice, which needs to be further examined.

## Introduction

Following its first conductancy in 1917, the oral glucose tolerance test (OGTT) has been a standard technique in experimental and clinical medicine and has particularly since 1980 been a key tool for diagnosis and screening of impaired glucose tolerance and type 2 diabetes (1). A key factor for the glucose tolerance after oral glucose is the augmentation of the insulin response by the incretin hormones, glucose-dependent insulinotropic polypeptide (GIP) and glucagon-like peptide-1 (GLP-1) (2–4). In fact, GIP and GLP-1 have been suggested to be responsible for approximately 60-70% of the insulin response after oral glucose, as judged from studies evaluating the difference in insulinemia after intravenous *versus* oral glucose administration at matched glucose levels both in humans (5) and animals (6).

Mice with genetic deletion of GIP receptors or GLP-1 receptors have been used to examined the impact on the two incretin hormones (7). To study the reported impact on glucose and insulin responses to oral glucose in these mice, we reviewed the literature in which studies on oral glucose challenges in GIP receptor KO or GLP-1 receptor KO mice have been performed. We found such studies reported in several studies (8–14) but that the clear role of the incretin hormones to maintain normal glucose and insulin responses to oral glucose was not entirely consistent, mainly because the responses were in general studied after a single dose of glucose administration. We have, furthermore, previously performed a stepwise oral glucose administration in mice with double genetic deletion of both GIP and GLP-1 receptors (DIRKO mice) (15). That study showed that these mice have glucose intolerance with impaired increase in insulin levels only after a large glucose load (75 and 100mg), but not after smaller glucose loads (25 and 50mg). A glucose dependent nature of the impact of genetic deletion of GIP and GLP-1 receptors on glucose and insulin responses to oral glucose may therefore exist, which may explain the partial inconsistency in the literature. To test this hypothesis we have, in the present study, therefore undertaken a study on the glucose and insulin responses to stepwise oral glucose administration over a wide range in GIP receptor KO and GLP-1 receptor KO mice.

## Methods

### Literature Survey

Key words for GIP receptor KO mice and GLP-1 receptor KO mice combined with oral glucose, glucose tolerance and insulin secretion were introduced in the PubMed data base. The found studies were read in detail in regard to methodology (gender and age of mice, length of fasting and oral glucose load, number of animals in each study arm, and results on glycemia and insulinemia).

### Animals

The generation of GLP-1 receptor KO mice and GIP receptor KO mice has been described previously (14). Briefly, mice on a C57BL6J background being heterozygous for the deletion of both the *Glp1r* and *Gipr* genes were generated from double homozygous deletion mutant mice by rederivation at Taconic Europe (Silkeborg, Denmark). Heterozygotes were mated to yield GLP-1 receptor KO mice, GIP receptor KO mice, and wildtype mice. The resulting offspring was used to establish breeding pairs, whose offspring was used in the experiments. All experiments were undertaken in female mice of 4–6 months of age. The animals were maintained in a temperature-controlled room (22°C) on a 12:12 h light-dark cycle (light on at 7:00 AM). Mice were fed a standard pellet diet (total energy 14.1 MJ/kg with 14% from fat, 60% from carbohydrate and 26% from protein; SAFE, Augy, France) and tap water ad libitum. During experimental days, food was removed from the cages at 7:30 AM and the actual experiments started at 12:30, i.e., during the light cycle. We used female mice only to avoid the stress of single housing, which is used in male mice, and to be in line with the previous study in GIP receptor KO and GLP-1 receptor KO mice (16). We used the mice randomly during the estrous cycle. The study was approved by the Lund/Malmö Animal Ethics Committee (Approval No. 5.8.18-06417/2020) and performed according to Good Laboratory Practice.

### Animal Disposition

A total of 238 animals were allocated for experimental procedure (98 wildtype mice, 68 GIP receptor KO mice and 72 GLP-1 receptor KO mice). Studies were undertaken in batches of 6–8 mice on each experimental day by one experienced technician. In all individual experiments, animals from all individual subgroups were involved to avoid bias in different results on different days. **Table 1** shows the detailed number of animals in each of the study groups. All individual results from the completer population were included in the final analysis and statistics.

**Table 1:** Number of animals in each of the study groups in this project.

Glucose dose (mg/mouse)	Wildtype mice	GIP receptor KO mice	GLP-1 receptor KO mice
0	6	8	6
25	11	11	10
50	12	12	10
75	12	9	9
100	34	20	27
125	24	8	16

### Experiments

After a 5-h fast, mice were anesthetized with a fixed dose combination of fentanyl (0.02 mg/mouse)-fluanisone (0.5 mg/mouse) (Hypnorm^R^; Vetpharma, Leeds, UK) and midozalam (0.125 mg/mouse; Roche, Basel, Switzerland) and given glucose (25-125 mg per mouse, dissolved in saline) or saline alone (i.e., 0 mg glucose) in the stomach through a gastric tube (outer diameter 1.2 mm). Whole blood was sampled in heparinized pipettes from the intraorbital retrobulbar sinus plexus (40 µl) at 0, 15, 30 and 60 min. Plasma was separated by centrifugation and stored at -20°C until analysis for insulin.

### Assays

Glucose was detected with the glucose oxidase method using Accu Chek Aviva (Hoffman-La Roche, Basel, Switzerland). Insulin was determined by ELISA (Mercodia, Uppsala, Sweden). The intra-assay coefficient of variation (CV) of the method is 4% at both low and high levels, and the interassay CV is 5% at both low and high levels. The lower limit of quantification of the assay is 6 pmol/l.

### Data Analysis

Data are presented as means ± SEM. Areas under the curves (total AUCs) were calculated with the trapezoid rule using glucose and insulin levels throughout the 60 min study period. The relative increase in AUC_insulin_ by increasing the glucose load was estimated by calculating the ratio between AUC_insulin_ at each of the glucose loads divided by AUC_insulin_ after zero glucose. The beta cell response was estimated as AUC_insulin_ divided by AUC_glucose_. Fasting insulin sensitivity was determined by the HOMA-R analyses (baseline glucose in mmol/l times baseline insulin in mU/l divided by 22.5) (17).

### Statistical Analysis

Differences between experimental groups were determined using a two-way analysis of variance (ANOVA) followed by a Sidak’s multiple comparisons test. Baseline insulin levels were applied to the Kolmogorov-Smirnov test for test of normality and results showed that significance for non-normality was not reached (in wildtype mice P=0.068, in GIP receptor KO mice P=0.103 and in GLP-1 receptor KO mice P=0.094). For all analyses, statistical significance was defined as *P <*0.05 and data reported as means ± SEM. Analyses were carried out using SPSS, v. 27.

## Results

### Review of the Literature

A total of six publications were found in the PubMed data base, in which studies on glucose and insulin levels after oral glucose challenges in GIP receptor KO mice and/or in GLP-1 receptor KO mice were reported (8, 10–14). By dividing the studies into different study arms depending on gender, GIP receptor KO and GLP-1 receptor KO animals, a total of thirteen different study arms were reported in these six publications (**Table 2**). Six studies used both female and male mice, whereas two studies used only male mice. The age of the mice was in general 3-4 months. All studies used long fasting period (>16 hrs) and the number of animals in each arm did in most studies exceed six, but also low numbers of only four animals were reported in some study arms. All studies examined only a single amount of glucose load, which varied between 1 and 3 mg/g. All studies measured glucose levels at several time points after glucose administration and most of them also determined the area under the glucose curve as a measure. In contrast, insulin levels were reported at several time points after glucose challenge in only one study, whereas insulin levels were usually reported at a single time point after glucose.

**Table 2:** Studies reporting glucose and insulin responses to oral glucose in homozygous GIP or GLP-1 receptor KO mice compared with their wildtype counterparts.

Reference	Experimental groups	Number of animals	Age	Length of fasting (hrs)	Glucose dose (mg/g)	Change in glycemia *versus* wildtype mice	Change in insulinemia *versus* wildtype mice
**GIP receptor KO studies**							
Miyawaki et al. (11)	Male GIP receptor KO	6	8-12 weeks	16	2	20 and 30 min_glucose_ ↑	15 and 30 min_insulin_ ↓
	Male wildtype	4					
Pamir et al. (12)	Male GIP receptor KO	11	9-14 weeks	16	1	15 and 30 min_glucose_ ↑ ≈18%	20 min_insulin_ ↓ ≈45%
	Male wildtype	7					
Preitner et al. (13)	Female GIP receptor KO	9	3-4 months	16	3	AUC_glucose_ ↑≈50%	15 min_insulin_ ↓≈30%
	Female wildtype	11					
	Male GIP receptor KO	(12-19)*	3-4 months	16	3	AUC_glucose_ ↑≈30%	15 min_insulin_ ↓≈50%
	Male wildtype	(12-19)*					
Hansotia et al. (14)	Female GIP receptor KO	(4-14)*	9-15 weeks	16-18	1.5	AUC_glucose_ ↔	Not determined
	Female wildtype	(4-14)*					
	Male GIP receptor KO	(13-28)*	9-15 weeks	16-18	1.5	AUC_glucose_ ↔	10 min_insulin_ ↔
	Male wildtype	(4-14)*					
**GLP-1 receptor KO studies**							
Scrocchi et al. (8)	Female GLP-1 receptor KO	(n=10)*	6-8 weeks	18	1.5	20, 30 and 90 min_glucose_ ↑	Not determined
	Female wildtype						
	Male GLP-1 receptor KO	(n=7)*	6-8 weeks	18	1.5	All time points_glucose_ ↑	30 min_insulin_ ↓ ≈40%
	Male wildtype						
Scrocchi et al. (9)	Male GLP-1 receptor KO	n=5	3 months	14-16	1.5	Not shown	30 min_insulin_ ↓ ≈50%
	Male wildtype	n=5					
Pederson et al. (10)	Female and male GLP-1R KO	(10-21)*	5-16 weeks	16	1	30 min_glucose_ ↑	Not determined
	Female and male wildtype	(10-21)*					
Preitner et al. (14)	Female GLP-1receptor KO	6	3-4 months	16	3	AUC_glucose_ ↑≈25%	15 min_insulin_ ↓≈40%
	Female wildtype	11					
	Male GLP-1 receptor KO	(9-13)	3-4 months	16	3	AUC_glucose_ ↔	15 min_insulin_ ↔
	Male wildtype	(9-13)					
Hansotia et al. (14)	Female GLP-1 receptor KO	(4-14)*	9-15 weeks	16-18	1.5	AUC_glucose_ ↔	Not determined
	Female wildtype	(4-14)*					
	Male GLP-1 receptor KO	(13-28)*	9-15 weeks	16-18	1.5	AUC_glucose_ ↔	10 min_insulin_ ↔
	Male wildtype	(4-14)*					

*indicates “not reported in individual groups”. ↑indicates increase, ↓reduction and ↔no change versus wildtype.

Result showed significantly higher glucose levels after oral glucose in GIP or GLP-1 receptor KO arms compared to wildtype mice (i.e., glucose intolerance) in eight of the study arms (62%) but no difference between KO mice and wildtype mice in five study armss (38%). Of the seven study arms with male mice, four had glucose intolerance (57%) and three no difference (43%), whereas of the six study arms with female mice, four had glucose intolerance (67%) and two no difference (33%). In five study arms,a high glucose loads of 2 or 3 mg/g was given and in these studies, four groups with GIP or GLP-1 receptor KO mice had glucose intolerance whereas one arm had no difference in glucose, i.e., 80% of the studies showed glucose intolerance. In contrast, in the studies using a lower glucose load of 1 or 1.5 mg/g, four out of eight study arms (i.e., 50%) showed glucose intolerance in GIP or GLP-1 receptor KO mice. In regard to insulinemia, lower insulin response to oral glucose in GIP or GLP-1 receptor KO mice compared to wildtype mice were reported in some but not in all studies. Therefore, the compiled results are not entirely consistent although approximately two third of the arms show glucose intolerance or a low insulin response to oral glucose in GIP or GLP-1 receptor KO mice; gender does not seem to explain this difference but there is a trend for a stronger phenotype after high glucose load than after low glucose load.

### Glucose and Insulin Responses to Stepwise Oral Glucose Administration

The glucose and insulin levels were examined before and at 15, 30 and 60 min after the oral administration of glucose in the range of 0 to 125 mg in wildtype mice and in GIP and GLP-1 receptor KO mice. **Table 1** shows the number of animals in each of the study groups, and **Table 3** shows baseline levels in all mice in the three mice strains. It is seen that GLP-1 receptor KO mice had higher fasting glucose than wildtype and GIP receptor KO mice. Insulin levels were not significantly different between the wildtype and the KO mice, although there was a trend of higher baseline insulin levels in GIP receptor KO mice than in wildtype mice (P=0.06). Fasting insulin sensitivity, as estimated by HOMA-R, was significantly lower in GIP and GLP-1 receptor KO mice than in wildtype mice.

**Table 3:** Body weight and baseline glucose and insulin levels and HOMA-R after 5 hrs of fasting in wildtype mice and in GIP receptor KO and GLP-1 receptor KO mice.

	Wildtype mice (n=99)	GIP receptor KO mice (n=68)	GLP-1 receptor KO mice (n=78)
Body weight (g)	21.8 ± 0.2	22.1 ± 0.2	20.5 ± 0.2 (P<0.001)
Glucose (mmol/l)	7.8 ± 0.1	7.8 ± 0.1	9.5 ± 0.1 (P<0.001)
Insulin (pmol/l)	231 ± 9	284 ± 13 (P=0.069)	226 ± 13
Insulin sensitivity (HOMA-R)	11.6 ± 0.5	14.3 ± 0.7 (P=0.020)	14.0 ± 0.8 (P=0.040)

Means ± SEM are shown. P indicates probability level of random difference versus wildtype mice. n indicates number of animals.

**Figure 1:**
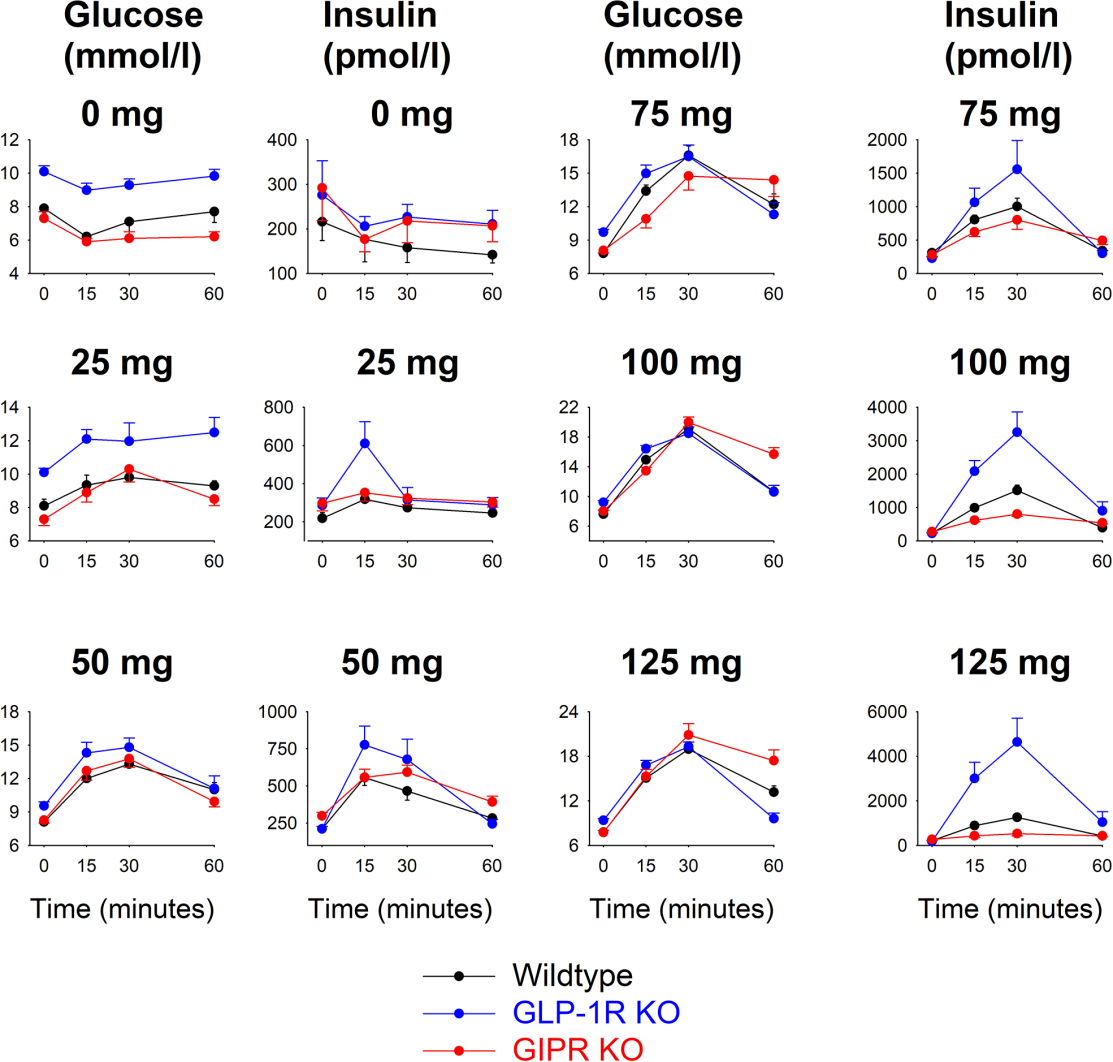
Glucose and insulin levels before and after oral administration of glucose at 0, 25, 50, 75, 100 or 125 mg in wildtype mice, GLP-1 receptor KO mice and GIP receptor KO mice. Means ± SEM are shown. There were 6-34 animals in each individual group (see **Table 1** for details). Observe that y-axis for the respective panels have been adjusted for the actual levels and are therefore different for the different glucose loads.

**Figure 1** shows glucose and insulin levels in all individual groups. After saline alone (i.e., 0 mg glucose), glucose and insulin levels were stable throughout the 60 min study period with no significant difference between the time points in any of the groups. Glucose levels remained higher from baseline throughout the 60 min period in GLP-1 receptor KO mice comparesd to wildtype mice. After oral administraton of 25 mg glucose, there were no significant changes in glucose and insulin levels compared to fasting levels in any of the three groups. After administration of glucose at 50, 75, 100 and 125 mg, glucose and insulin levels rose in all three groups with peaks being observed after 30 min.

**Figure 2:**
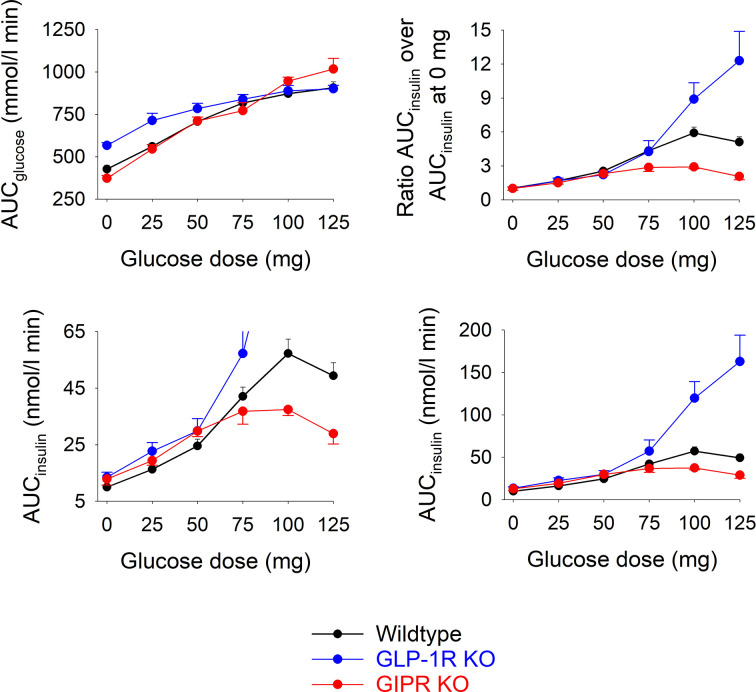
Upper left panel: Total AUC_glucose_ after oral administration of glucose at 0, 25, 50, 75, 100 or 125 mg in wildtype mice, GLP-1 receptor KO mice and in GIP receptor KO mice. Upper right panel: Ratio of total AUC_insulin_ after oral administration of glucose at 0, 25, 50, 75, 100 or 125 mg divided by total AUC_insulin_ after saline alone (i.e., zero glucose) in wildtype mice, GLP-1 receptor KO mice and in GIP receptor KO mice. Lower panels: Total AUC_insulin_ after oral administration of glucose at 0, 25, 50, 75, 100 or 125 mg in wildtype mice, GLP-1 receptor KO mice and in GIP receptor KO mice; observe two different ranges in y-axis. Means ± SEM are shown. There were 6-34 animals in each individual group (see **Table 1** for details).

**Figure 2** shows total AUC_glucose_ and total AUC_insulin_ during the 60 min test after each of the glucose doses in the three groups of mice.). In wildtype mice, AUC_glucose_ increased by increasing the glucose load up to 75 mg, whereas at higher glucose levels, there was no further increase in AUC_glucose_. In GIP receptor KO mice, AUC_glucose_ increased by increasing the glucose load with no trend of leveling off at the highest glucose. AUC_glucose_ did not differ in GIP receptor KO mice from wildtype mice after 25, 50, 75 and 100 mg glucose, whereas AUC_glucose_ was higher in GIP receptor KO mice than in wildtype mice after 125 mg (P=0.029). GLP-1 receptor KO mice had higher AUC_glucose_ than wildtype mice after zero (P<0.001) and 25 mg glucose administration (P=0.007), but with no difference at higher glucose doses. Also AUC_insulin_ increased gradually in wildtype mice by increasing the gucose load up to 100 mg. In GIP receptor KO mice, AUC_insulin_ was significantly lower than in wildtype mice after 100 mg glucose (P=0.003) and 125 mg glucose (P=0.001), but not after lower glucose doses. In GLP-1 receptor KO mice, AUC_insulin_ was higher than in wildtype mice after 100 mg glucose and 125 mg glucose (both P<0.001) but not at lower glucose doses.

As a marker of the relative increase in AUC_insulin_ by increasing the glucose load, the ratio between AUC_insuin_ at each of the glucose doses and the AUC_insulin_ after zero glucose was calculated (**Figure 2**). In GIP receptor KO mice, this ratio was significantly lower than in wildtype mice after 100 mg glucose (P=0.007) and after 125 mg (P=0.008). In contrast, the ratios were significantly higher in GLP-1 receptor KO mice than in wildtype mice after 125 mg (P=0.032).

**Figure 3:**
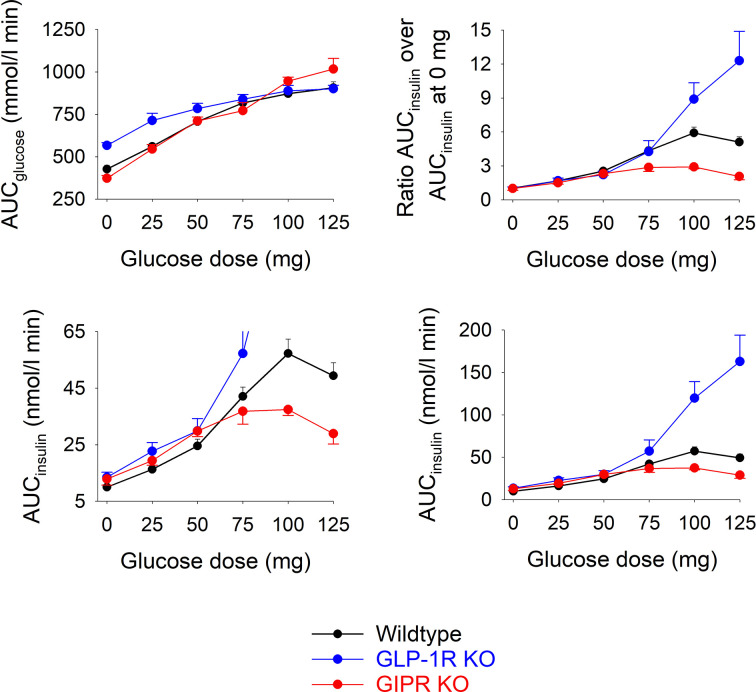
Beta cell response (AUC_insulin_ divided by AUC_glucose_) after oral administration of glucose at 0, 25, 50, 75, 100 or 125 mg in wildtype mice, GLP-1 receptor KO mice and GIP receptor KO mice. Means ± SEM are shown. There were 6-34 animals in each individual group (see **Table 1** for details).

As a marker of the beta cell response to oral glucose, AUC_inusulin_ was divided by AUC_glucose_ at each of the glucose loads (**Figure 3**). It is seen that this marker was lower in GIP receptor KO mice than in wildtype mice after 100 mg (P=0.036) and 125 mg (P=0.018), and higher in GLP-1 receptor KO mice than in wildtype mice after 100 mg (P=0.029) and 125 mg (P<0.001).

## Discussion

We reviewed the literature on glucose and insulin responses to oral glucose in GIP and GLP-1 receptor KO mice and found that glucose intolerance is not as robustly demonstrated as is generally assumed. In fact, only approximately two thirds of the study arms with GIP receptor or GLP-1 receptor KO mice *versus* wildtype mice showed glucose intolerance accompanied by a reduced insulin response, whereas in approximately a third of study arms these KO mice had normal glucose tolerance (8, 10–14). The explanation may be that the phenotype varies between different KO colonies, but differences in the experimental approaches may also explain the incosistencies. A general limitation of the studies was that they used a long semistarvation fasting period of >16 hours and only one single glucose load was used in each study. There was no clear differences in the background data in studies reporting glucose intolerance or normal glucose tolerance in GIP or GLP-1 receptor KO mice, although a trend of a more severe glucose intolerance was reported at higher glucose loads. Thus, studies on glucose tolerance after oral glucose administration in GIP and GLP-1 receptor KO mice show a trend but not a general consistency of glucose intolerance. The trend of differences in results depending on glucose loads prompted the present study of a full-range stepwise glucose administration design in GIP and GLP-1 receptor KO mice.

The stepwise dose-response study evaluated glucose and insulin responses to glucose challenges ranging from 25 to 125 mg in wildtype, GIP and GLP-1 receptor KO mice. Using this large range of glucose allows conclusions after both low and high glucose. Other strengths of the study are that we also included a group with zero glucose administration and that we used mice with a physiological fasting period of five hours, which is in the range of previously reported optimal period for mice in studies on glucose tolerance (18). We also standardized the mice in age of the animals and all experimental studies were performed at the same time of the day and by the same experienced technician. We also included wildtype mice together with receptor KO mice in each batch of study animals, which is important considering the day-to-day variations in metabolism.

We found that in wildtype mice, glucose levels increased by increasing the glucose load up to the 75 mg dose, thereafter no further increase in glucose levels occur in spite of larger glucose loads. At the same time, insulin levels also increased by increasing the oral gucose load up to 75 mg. This together suggests that there is a saturation of glycemia after oral glucose in normal physology in mice and that this is achieved by increasing insulin levels. This has previously been shown also in humans (4) and is ascribed to increased insulin due to glucose in combination with the incretin effect.

The GIP receptor KO mice had normal glucose tolerance compared to wildtype mice after 25 and 50 mg. In contrast, the GLP-1 receptor KO mice had higher AUC_glucose_ than the other groups after 25 and 50 mg, but this was mainly related to the higher fasting glucose, which most likely is a phenomenon explained by exaggerated hepatic glucose production due to absence of GLP-1 action to inhibit liver glucose production, which has been demonstrated in rodents (19) and humans (20, 21) and which may be mediated by insulin and glucagon, but also mediated by GLP-1 receptors located in the portal system (22, 23). Nevertheless, the increase in glucose levels after oral glucose was similar in GLP-1 receptor KO mice as in the other groups, but from a higher levels. These data together suggest that GLP-1 receptor and GIP receptor KO mice maintain glucose tolerance after low glucose loads, which supports the trend in the literature that glucose intolerance is rather seen after high glucose loads than after low glucose loads in GIP or GLP-1 receptor KO mice (**Table 2**).

When glucose loads were raised to 100 and 125 mg, glucose levels continued to increase in GIP receptor KO mice in comparison to lower glucose loads, whereas this was not observed in GLP-1 receptor KO mice. This is similar as our previous results in DIRKO mice, which have both GIP and GLP-1 receptor gene deletion (15), whichs suggests that GIP rather than GLP-1 explains the glucose intolerance after high oral glucose loads in DIRKO mice.

The reason why GIP receptor KO mice had glucose intolerance at higher glucose levels seems to be explained by the failure to further increase insulin levels; in fact, at the high glucose load AUC_insulin_ was increased only approximately twofold in GIP receptor KO mice compared to zero glucose administration, whereas it was increased six times in wildtype mice. In contrast, GLP-1 receptor KO mice had an increase in the insulin response to oral glucose at the high glucose loads of 100 and 125 mg. In fact, AUC_insulin_ increased more than twelve-fold at 125 mg *versus* zero glucose compared to six-fold in wildtype mice; this high insulin is most likely the reason that glucose levels were prevented from being increased further. The nature of this compensatory mechanism in GLP-1 receptor KO mice at high oral glucose load is not known but deserves further studies – candidates may be the higher baseline glucose levels or compensatory increase in other gut hormones which may mask the consequence of GLP-1 receptor gene deletion. Studies on these adaptive mechanisms are important future development of the present study, since the mechanisms may involve important novel regulatory mechanisms for islet function and glucose tolerance. This may be of relevance also for human physiology and pathophysiology of type 2 diabetes.

Although not within the aim of this study, beta cell function may be indirectly determined by comparing the increase in AUC_insulin_ after each of the glucose loads to AUC_insulin_ after zero glucose. This was six times higher at 100 mg glucose than at zero glucose in wildtype mice, significantly reduced in GIP receptor KO mice and enhanced in GLP-1 receptor KO mice. Beta cell activity was also indirectly determined by dividing the increase in insulin levels by the increase in glucose levels at 30 min after glucose administration. It was found that this marker of beta cell function increased by increasing the glucose load in wildtype mice up to 100mg. In GIP receptor KO mice, beta cell failure was evident after 100 and 125mg glucose, whereas an adaptive increase in beta cell function was seen in GLP-1 receptor KO mice.

Although the studies have several strengths, of which the use of a full-range stepwise glucose administration design is most important, there are also several limitations with these studies. First, GIP receptor and GLP-1 receptor deletion may result in adaptive responses, which may prevent conclusions on the individual contributions by GIP and GLP-1, and the increase in the insulin response to oral glucose in GLP-1 receptor KO mice is such an adaptation. Further studies using specific pharmacological blockade are therefore warranted. Second, we studied only female mice, and therefore our conclusions are valid only for this gender. Third, we did not determine other potential incretin hormones or glucagon in this study; glucagon secretion in GIP and GLP-1 receptor KO mice has been examined in previous studies (16).

In conclusion, we have performed a literature survey of studies reporting glucose and insulin levels after oral glucose in GIP receptor or GLP-1 receptor KO mice and found that glucose intolerance is evident in approximately two thirds of study arms, whereas normal glucose tolerance is evident in one third of study arms. We have also performed a complete dose study by delivering glucose loads from zero to 125 mg to female wildtype and GIP receptor KO and GLP-1 receptor KO mice. We have demonstrated 1) that glucose levels saturate at levels above 75 mg in wildtype mice due to increase in insulin levels, 2) that GIP receptor KO mice have glucose intolerance after high glucose load due to insufficient increase in insulin levels, and 3) that GLP-1 receptor KO mice maintain glucose tolerance in spire of having GLP-1 receptor gene deletion due to a compensatory increase in insulin levels.

## Data Availability Statement

The raw data supporting the conclusions of this article will be made available by the authors, upon reasonable request.

## Ethics Statement

The animal study was reviewed and approved by Lund/Malmö Animal Ethics Committee (Approval No. 5.8.18-06417/2020).

## Author Contributions

BA: Conceptualization, Data curation, Formal analysis, Funding acquisition, Investigation, Methodology, Project administration, Resources, Software, Supervision, Validation, Writing - original draft, Writing - review & editing. YY: Writing - review and editing. YS: Writing - review and editing. All authors contributed to the article and approved the submitted version.

## Funding

This research was funded by the Lund University Medical Faculty, Region Skåne, and the Swedish Research Council (to B. Ahrén).

## Conflict of Interest

The authors declare that the research was conducted in the absence of any commercial or financial relationships that could be construed as a potential conflict of interest.
